# Post-Transcriptional Modifications to miRNAs Undergo Widespread Alterations, Creating a Unique Lung Adenocarcinoma IsomiRome

**DOI:** 10.3390/cancers16193322

**Published:** 2024-09-28

**Authors:** David E. Cohn, Vanessa G. P. Souza, Aisling Forder, Nikita Telkar, Greg L. Stewart, Wan L. Lam

**Affiliations:** 1 Department of Integrative Oncology, BC Cancer Research Institute, Vancouver, BC V5Z 1L3, Canadawanlam@bccrc.ca (W.L.L.); 2 British Columbia Children’s Hospital Research Institute, Vancouver, BC V5Z 4H4, Canada

**Keywords:** isomiR, miRNA, lung adenocarcinoma, ADAR, RNA editing

## Abstract

**Simple Summary:**

MicroRNAs (miRNAs) play a significant role as epigenetic regulators in cancer. IsomiRs are miRNA molecules that undergo small modifications during miRNA processing, which can affect their stability and their interaction with mRNA targets. While some isomiRs are linked to specific cancers, many, including those in the lung, remain understudied. To address this, small RNA sequencing data from lung adenocarcinoma (LUAD) and adult non-malignant lung (ANL) samples were analyzed to quantify isomiR expression. This analysis identified 16 A-to-I edited isomiRs, 213 5′ isomiRs, 128 3′ adenylated isomiRs, and 100 3′ uridylated isomiRs. A-to-I editing rates correlated with the expression of editing enzymes ADAR and ADARB1, both deregulated in LUAD. LUAD samples had lower A-to-I editing and 3′ adenylation rates compared to ANL. Machine learning models based on isomiR data effectively distinguished ANL from stage I/II LUAD samples, suggesting that isomiRs hold potential as cancer biomarkers.

**Abstract:**

Background: MicroRNAs (miRNAs) modulate the expression of oncogenes and tumor suppressor genes, functioning as significant epigenetic regulators in cancer. IsomiRs are miRNA molecules that have undergone small modifications during miRNA processing. These modifications can alter an isomiR’s binding stability with mRNA targets, and certain isomiRs have been implicated in the development of specific cancers. Still, the isomiRomes of many tissues, including the lung, have not been characterized; Methods: In this study, we analyzed small RNA sequencing data for three cohorts of lung adenocarcinoma (LUAD) and adult non-malignant lung (ANL) samples. Results: We quantified isomiR expression and found 16 A-to-I edited isomiRs expressed in multiple cohorts, as well as 213 5′ isomiRs, 128 3′ adenylated isomiRs, and 100 3′ uridylated isomiRs. Rates of A-to-I editing at editing hotspots correlated with mRNA expression of the editing enzymes ADAR and ADARB1, which were both observed to be deregulated in LUAD. LUAD samples displayed lower overall rates of A-to-I editing and 3′ adenylation than ANL samples. Support vector machines and random forest models were trained on one cohort to distinguish ANL and stage I/II LUAD samples using reads per million (RPM) and frequency data for different types of isomiRs. Models trained on A-to-I editing rates at editing hotspots displayed high accuracy when tested on the other two cohorts and compared favorably to classifiers trained on miRNA expression alone; Conclusions: We have identified isomiRs in the human lung and found that their expression differs between non-malignant and tumor tissues, suggesting they hold potential as cancer biomarkers.

## 1. Introduction

MicroRNAs (miRNAs) are a highly expressed subtype of endogenous small non-coding RNAs that canonically downregulate genes through binding to partially complementary regions of mRNA and either inhibiting translation or inducing mRNA degradation [1,2,3]. miRNA dysregulation is a common mechanism through which tumors deregulate critical pathways, and many miRNAs are oncogenes or tumor suppressor genes for certain cancer types [4]. Furthermore, the small size of miRNAs renders them highly stable, and consequently, any miRNAs that are deregulated in tumors are appealing candidate diagnostic biomarkers [5]. These factors have driven interest in the characterization of tumor miRNomes, particularly for cancers such as lung adenocarcinoma (LUAD), which often presents without actionable driver mutations [6].

However, the study of the miRNomes of LUAD and other tumors has been complicated by the fact that most miRNA-coding loci generate not only an unmodified (“canonical”) miRNA but also distinct modified miRNA molecules known as isomiRs [7]. The sequence modifications that distinguish isomiRs arise from a variety of sources, including the cleavage of miRNA precursor molecules by Drosha or Dicer at non-canonical sites, the adenosine deaminase activity of ADAR-family (adenosine deaminase, RNA-specific) proteins, and the activity of terminal nucleotidyltransferase proteins [7] (Figure S1). IsomiRs typically interact with gene-silencing machinery in the same way as canonical miRNAs [8], but their sequence changes mean that they do not necessarily target the same mRNAs [9].

The ubiquity of miRNA deregulation in cancer has motivated research into whether tumors also frequently alter the expression of isomiRs. Several adenosine-to-inosine (A-to-I) edited isomiRs are dysregulated in various forms of cancer, causing downstream changes in gene expression that benefit tumors [10,11]. Different tissues are known to have distinct patterns of isomiR expression [12], which suggests that tumorigenesis in different tissues is likely associated with unique changes to their isomiR transcriptomes (isomiRomes). The characterization of the isomiRomes of specific tumors, such as LUAD, is thus of interest, especially as miRNA deregulation has already been extensively tied to LUAD tumor growth and therapeutic resistance [13,14,15,16,17,18]. Understanding how the lung isomiRome is altered in LUAD will provide further insight into how LUAD tumors orchestrate and benefit from miRNA deregulation and could enable the improvement of existing miRNA-based biomarker panels through the incorporation of informative isomiRs.

RNA modifications, including A-to-I editing, are associated with a range of lung diseases, particularly lung cancer [19]. Investigations into A-to-I edited isomiRs have utilized data from The Cancer Genome Atlas (TCGA), a major and comprehensive cancer genomics resource [20]. These studies have pinpointed editing sites that distinguish between adult non-malignant lung (ANL) and lung adenocarcinoma (LUAD) tissues and have linked these sites to patient survival [21,22]. Further validation was provided by a TCGA lung-specific study, which identified distinct mRNA targets for edited isomiRs [23]. Additionally, A-to-I edited isomiRs were detected in the British Columbia Cancer Agency (BCCA) lung cohort [24]. These findings were corroborated by a Japanese lung cohort study, which found that reduced editing of miR-99a was associated with patient survival [24].

While these studies have made significant contributions to our understanding of isomiRs in lung cancer, there are areas where further investigation could enhance this understanding. Many of these studies primarily relied on TCGA data, and while TCGA data are comprehensive, validation across additional diverse patient groups is important, given that isomiR expression can vary based on patient characteristics such as race [25]. Moreover, the focus has largely been on A-to-I edited isomiRs, leaving other major types, such as 5′ modified, 3′ adenylated, and 3′ uridylated isomiRs, less explored in the context of lung cancer. Understanding these isomiRs is crucial, as they may have unique functions that could provide deeper insights into disease mechanisms [25]. For example, 5′ modified isomiRs may target different mRNAs compared to their canonical counterparts [26], while 3′ uridylated and adenylated isomiRs have been shown to influence gene regulation and miRNA stability [27,28,29].

To expand on earlier discoveries, we conducted a comprehensive study examining the different types of isomiRs across three distinct lung cohorts. We identified differences in isomiR expression between ANL and LUAD tissues, both overall and at the individual isomiR level. Additionally, we investigated potential causes of these differences by comparing the expression level of key miRNA-modifying genes between ANL and LUAD and analyzing their correlations with isomiR expression. Furthermore, we explored the relationship between isomiR expression and patient outcomes and assessed the potential of isomiRs as biomarkers for stage I/II LUAD.

## 2. Materials and Methods

### 2.1. Sample Collection, Sequencing, and Data Processing

Three lung cohorts were used in this study: the British Columbia Cancer Agency (BCCA) internal cohort containing ANL and LUAD samples, The Cancer Genome Atlas LUAD external cohort (TCGA-LUAD, hereafter TCGA) of ANL and LUAD samples, and the Ewha Womans University (EWU) external cohort, which consists of pairs of ANL and LUAD samples. Clinical characteristics are presented in Table 1.

For mRNA expression data, a total of 76 pairs of ANL and LUAD samples within the BCCA cohort were available for the study [30]. However, fewer LUAD samples (*n* = 63) were sequenced for the small RNA data. The 76 paired ANL and LUAD samples were obtained with informed and written consent from patients at Vancouver General Hospital and with approval from the University of British Columbia/BC Cancer Agency Research Ethics Board (H15-03060), as previously described [31]. After reviewing the histology of the tissue samples, a lung pathologist guided the microdissection process to ensure that ANL and LUAD samples had >80% non-malignant and tumor cell content, respectively. Following this, TRIzol reagent was used to extract total RNA from the frozen sections of samples of the BCCA cohort (Life Technologies, Carlsbad, CA, USA). The small RNA sequencing protocol on the Illumina HiSeq2000 platform (Illumina Inc., San Diego, CA, USA) at Canada’s Michael Smith Genome Sciences Centre has been previously described [32]. Small RNA sequencing files were obtained as Binary Alignment Map (BAM) files and converted to unaligned FASTQ files [32]. miRNA expression data for samples in the BCCA cohort were deposited in the National Center for Biotechnology Information Gene Expression Omnibus (GSE175462) [33]. The mRNA expression data for 76 pairs of ANL and LUAD samples in the BCCA cohort were generated using HumanWG-6 Gene Expression BeadChips (Illumina Inc., San Diego, CA, USA), as previously described [30]. Data downloaded from Ensembl Release 104, using Ensembl BioMart [34], were used to convert between probe names and the corresponding Ensembl gene stable IDs or gene names, where necessary [35]. In cases where a gene had multiple matching probes, the probe corresponding to the highest mean expression level was used.

TCGA miRNA sequencing data were available for 38 ANL samples and 395 LUAD samples, representing 389 LUAD patients after averaging multiple tumor sampling. mRNA sequencing data for the TCGA cohort were accessible for 59 ANL samples and 513 LUAD patients. After averaging multiple tumor samples, 57 paired samples were identified. All data were downloaded as Binary Alignment Map (BAM) files from the Genomic Data Commons Data Portal (https://portal.gdc.cancer.gov/, accessed on 11 July 2019). The TCGA samples were sequenced on the Illumina HiSeq2500 platform, as previously described [36]. mRNA expression data and patient clinical information, including survival data, were obtained from the University of California Santa Cruz (UCSC) Xena browser (https://xenabrowser.net/, accessed on 11 July 2019).

The EWU external cohort consists of 48 pairs of ANL and LUAD samples collected at the Samsung Medical Center. miRNA sequencing data for the EWU cohort were downloaded as FASTQ files from the European Nucleotide Archive (Study Accession: PRJNA434883) (https://www.ebi.ac.uk/ena/browser/home, accessed on 12 February 2021) [37]. All EWU samples were sequenced on the Illumina HiSeq2000 platform [37]. Clinical data for this cohort were obtained through the Sequence Read Archive run selector (SRA Accession: SRP133217) (https://www.ncbi.nlm.nih.gov/sra, accessed on 12 February 2021). mRNA expression and survival data were not publicly available. Samples with low coverage (<5 million reads) were excluded from all miRNA- and isomiR-level analyses for all cohorts.

### 2.2. Processing of Small RNA Sequencing Data

The 3′ adapter sequences were trimmed from the EWU cohort FASTQ files using the same algorithm that had been applied to the BCCA and TCGA cohorts (described in [36]). The algorithm was modified to accept FASTQ files as input. For all three cohorts, reads with any positions at which the Phred quality score was below 25 were removed using the FASTX-Toolkit (http://hannonlab.cshl.edu/fastx_toolkit/, accessed on 1 March 2022). Adapter-trimmed, quality-filtered files were then processed by miRMaster 2.0, which aligned reads to the hg38 assembly and quantified isomiR expression [38]. All default settings were used, apart from the following changes: Protocol = Custom; 3′ Adapter = ATCTCGTATGCCGTCTTCTGCTTGT (for the BCCA and TCGA cohorts) or TGGAATTCTCGGGTGCCAAGG (for the EWU cohort); Minimum read length = 15; Sliding window required quality = 1; Alignment tool = Bowtie; Maximum allowed distance for 3′ quantification = 2; and Minimum read stack height = 100. Following miRMaster processing, isomiRs with multiple substitutions relative to their corresponding mature miRNA were discarded.

### 2.3. IsomiR and miRNA Quantification and Nomenclature

For each type of isomiR being studied (5′ modified, 3′ adenylated, 3′ uridylated, and A-to-I edited), isomiRs of the same miRNA that differed only in non-relevant positions were combined. For example, when quantifying 5′ isomiRs, isomiRs that had identical 5′ nucleotide additions or deletions were combined, regardless of their 3′ variation. Similarly, when quantifying 3′ adenylated or uridylated isomiRs, isomiRs that had the same 3′ non-templated addition (NTA) were combined, regardless of any 5′ variation. Additionally, for consistency with past studies of miRNA editing, the final two bases of each isomiR were ignored when quantifying edited isomiRs,, and edited isomiRs that differed only in those positions were combined [39]. miRNA expression was quantified by combining the reads corresponding to each canonical miRNA with those of all isomiRs that corresponded to the miRNA. All isomiR names begin with the name of their corresponding canonical miRNA. For 5′ isomiRs, the miRNA name is then followed by a number indicating the position of the isomiR’s 5′ end relative to the miRNA’s 5′ end. A-to-I edited isomiR names have the same format, with an additional number that indicates the position of the edited adenosine (with 0 corresponding to the isomiR’s first nucleotide). 3′ adenylated and uridylated isomiR names contain a single number indicating the position of the adenylation (either 1 or 2) relative to the miRNA’s 3′ end, followed by an “A” or “U”.

### 2.4. Identification of High-Confidence miRNAs and isomiRs

First, isomiRs with substitutions or non-templated additions (NTAs) that matched single nucleotide polymorphisms listed in miRNASNP-v3 (http://bioinfo.life.hust.edu.cn/miRNASNP/, accessed on 1 August 2021) [40] or that matched lung cancer somatic mutations listed in COSMIC v94 (https://cancer.sanger.ac.uk/cosmic, accessed on 1 August 2021) [41] were discarded. For an isomiR to be considered expressed in a given sample group, it had to meet two criteria, each in ≥10% of the group’s samples: (a) expression at ≥ 1 RPM and (b) expression at a sufficiently high rate, relative to similar sequences, to distinguish it from a sequencing error. miRNAs were considered expressed if they met criterion (a) only. Criterion (b) was applied using a widely used method first described by Alon et al. for the detection of miRNA editing sites: briefly, the count of an isomiR’s reads and the count of otherwise identical reads that lacked the isomiR’s characteristic edit/3′ NTA/5′ variation were input into a binomial cumulative distribution, which used the baseline sequencing error rate to estimate the likelihood that all of the isomiR’s reads had arisen from errors when sequencing similar sequences [39]. This sequencing error hypothesis was rejected in a given sample if this likelihood was <0.05 after Benjamini–Hochberg (BH) correction and there were at least three reads of the isomiR in question. Because small RNA sequencing data are prone to plate-based batch effects [42], the baseline sequencing error rate for substitutions was taken as the greater of the expected rate (Phred 25 = 0.32%) and the observed rate of mismatches of the same type in the same position of a read in samples sequenced on the same plate. For 5′, 3′ adenylated, and 3′ uridylated isomiRs, the error rate was very conservatively set to 2%.

Expressed isomiRs were then discarded if they cross-mapped to other regions of the genome, as this indicated that their sequences may not have arisen through modification of a canonical miRNA transcript. Specifically, an isomiR was discarded if its most highly expressed sequence mapped, using Bowtie [43], to any other genomic site as well as it mapped to the genomic region of its miRNA stem-loop. Since transfer RNAs (tRNAs) receive post-transcriptional 3′ additions of CCA [44], isomiRs ending in CCA were also discarded if they cross-mapped to a tRNA-coding genomic region after the removal of those three bases. Finally, an isomiR or miRNA was deemed high-confidence if it was expressed in at least one of the two sample groups (ANL or LUAD) for at least two of the three adult lung cohorts.

### 2.5. Computation of Sample-Wide miRNA Editing/Adenylation/Uridylation Rates

The frequency of an isomiR was defined as the ratio of its expression to the combined expression of its corresponding canonical miRNA and all of that miRNA’s isomiRs. For each sample, a z-score was calculated for each high-confidence isomiR, indicating how its frequency in that sample varied in relation to its frequency in all samples from all three cohorts. The overall rate of a miRNA modification (e.g., 3′ adenylation) in a sample was then calculated as the mean z-score for all high-confidence isomiRs of that type in that sample. IsomiRs with an undefined z-score in certain samples, due to those samples having no expression of either the isomiR or the corresponding miRNA, were excluded when the mean z-score for those samples was calculated.

### 2.6. Determination of Significant Differences between ANL and LUAD Samples

Paired-sample *t*-tests were used to assess whether various quantities (i.e., the expression of miRNA-modifying enzymes or the overall rates of miRNA modifications) in each cohort differed between paired ANL and LUAD samples, with a significance threshold of *p* < 0.05. When determining whether the frequencies of individual isomiRs differed between ANL and LUAD samples, a false discovery rate (FDR) threshold < 0.05 after BH correction was used instead.

### 2.7. Analysis of Correlations between Variables

Correlations between the expression of miRNA-modifying enzymes and the rates of miRNA modifications or the frequencies of specific isomiRs were evaluated only in LUAD samples. A correlation was considered significant if Spearman’s ρ was different from zero, with a BH-FDR threshold of <0.05.

### 2.8. Survival Analyses

For all survival analyses, LUAD patients in the analyzed cohort were divided into groups based on having above-median or below-median values of the quantity being analyzed. The quantity was deemed to impact outcomes if the difference in overall survival between the two groups was significant, as assessed by a log-rank test (*p* < 0.05).

### 2.9. Support Vector Machine and Random Forest Classifiers

All classifiers were trained and tested only on ANL and stage I/II LUAD samples, using either miRNA RPM values or either RPM or frequency values for a single type of isomiR. All data were standardized for support vector machine (SVM) classifiers, and 300 trees were grown for each random forest (RF) classifier. isomiRs that had undefined frequencies in any training or testing sample were excluded from frequency-based classifiers.

For classifiers designed to discriminate between ANL and LUAD samples from a single cohort, data for all miRNAs or isomiRs of the chosen type that were expressed in the cohort were used, including those that were not high-confidence. The classification error for SVMs was calculated as the mean classification error of trained models during 10-fold cross-validation (CV), and the classification error for RFs was calculated as the ensemble out-of-bag error.

Classifiers that were designed to test the generalizability of isomiR-based biomarkers were always trained on the largest (TCGA) cohort. Data were used for all miRNAs or isomiRs of the chosen type that were expressed in the TCGA cohort, and that were differentially expressed (for RPM-based classifiers) or differentially frequent (for frequency-based classifiers) between TCGA ANL and TCGA stage I/II LUAD samples. Model hyperparameters were optimized using MATLAB’s Bayesopt function. BoxConstraint and KernelScale were optimized for SVMs by minimizing the mean classification error during a 5-fold CV. For the RFs, the minimum leaf size and the percentage of features that were made available to the classifier at each node were optimized, with maximum values of 20 and 70%, respectively, by minimizing the out-of-bag error. Trained, optimized models were tested on the BCCA and EWU cohorts.

### 2.10. Statistical and Graphical Software

All statistical analysis was performed in MATLAB R2017b (MathWorks, Natick, MA, USA). Except where otherwise specified, all illustrations were also generated in MATLAB.

## 3. Results

### 3.1. Characterization of the Lung isomiRome

**Figure 1:**
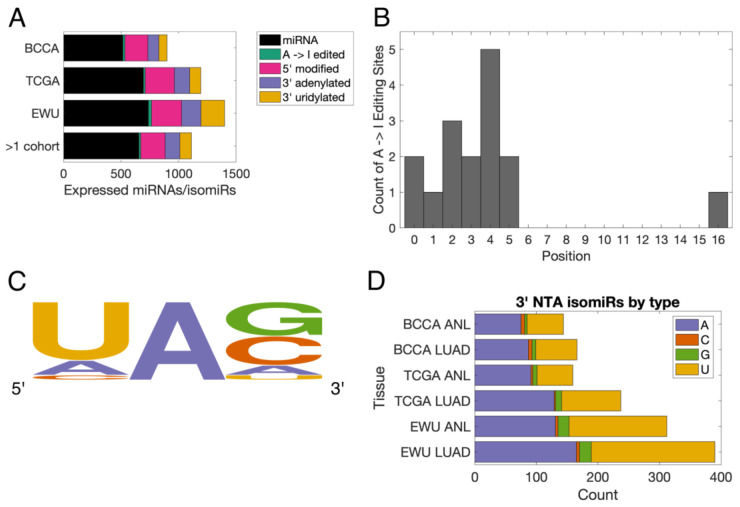
Basic characteristics of the lung isomiRome. (**A**) Bar chart illustrating the numbers of miRNAs and different types of isomiRs that were expressed in each lung cohort. The bottom row indicates the numbers of high-confidence miRNAs/isomiRs. The bar graph includes isomiRs that are expressed in ANL and LUAD samples in the individual cohorts. (**B**) Histogram indicating the positions of the 16 high-confidence A-to-I editing sites. Position ‘0’ corresponds to the first nucleotide of a miRNA. (**C**) Plot of the nucleotides located immediately 5′ and 3′ of the high-confidence editing sites, with the height of a letter being proportional to its frequency. Generated using WebLogo (v.2.8.2) [45]. (**D**) Bar chart indicating the numbers of isomiRs with 3′ non-templated addition (NTAs) of each nucleotide that was expressed in each sample group. ANL: Adult Non-malignant Lung; BCCA: British Columbia Cancer Agency; EWU: Ewha Womans University; LUAD: Lung Adenocarcinoma; TCGA: The Cancer Genome Atlas.

We found that similar numbers of 5′ isomiRs, 3′ adenylated isomiRs, and A-to-I edited isomiRs were expressed in each of the three lung cohorts, while there was an elevated number of 3′ uridylated isomiRs in the EWU cohort (Figure 1A). In total, there were 16 A-to-I edited isomiRs, 213 5′ isomiRs, 128 3′ adenylated isomiRs, 100 3′ uridylated isomiRs, and 654 miRNAs that were expressed in multiple cohorts, and thus deemed “high-confidence” (Figure 1A). Of these 16 edited isomiRs, 13 (81%) had editing sites that were located within their seed sequence (Figure 1B and Table 2). Twelve (75%) of the canonical miRNAs had an uracil immediately 5′ of the edited adenine, in line with the known sequence preferences of the editing enzymes ADAR and ADARB1 (Figure 1C and Table 2). IsomiRs with the 3′ NTA of cytosine or guanine were observed in all cohorts and tissue types, but they were far less common than 3′ adenylated and uridylated isomiRs (Figure 1D).

### 3.2. The Lung isomiRome Is Widely Altered in LUAD

**Figure 2:**
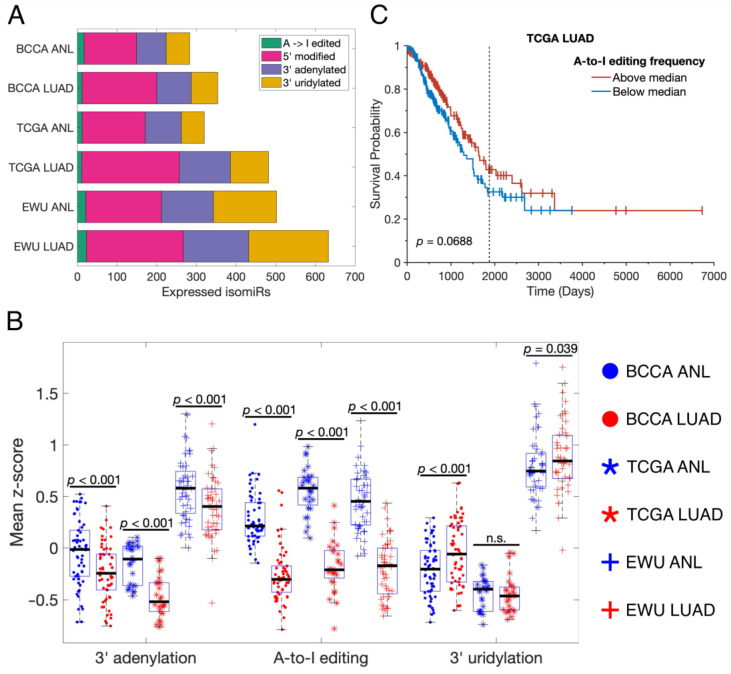
LUAD tumors alter the prevalence and rates of common miRNA modifications. (**A**) Bar chart showing the numbers of different types of isomiRs that were expressed in each sample group. (**B**) Scatter-box plots indicating the rates of three major types of miRNA modification in different sample groups, indicated by the legend on the right-hand side. Each point represents a single sample, and only paired ANL and LUAD samples were included. The displayed *p*-values resulted from paired-sample *t*-tests. n.s.: not significant. (**C**) Overall survival curves for TCGA LUAD patients for whom small RNA sequencing and clinical data were available (*n* = 379), stratified by their tumors’ A-to-I miRNA editing rates. The displayed *p*-value resulted from a log-rank test. The graphical representation was generated using MatSurv [47]. The vertical line indicates the five-year mark. ANL: Adult Non-malignant Lung; BCCA: British Columbia Cancer Agency; EWU: Ewha Womans University; LUAD: Lung Adenocarcinoma; TCGA: The Cancer Genome Atlas.

Upon comparing the ANL and LUAD isomiRomes, we found that LUAD samples expressed more isomiRs of all types, except A-to-I edited isomiRs, than ANL samples (Figure 2A). This suggests that the LUAD isomiRome is more diverse than that of ANL, which is consistent with past observations from pan-cancer datasets [46]. To explore differences in the rate of miRNA modifications, we defined the “frequency” of an isomiR to be the ratio of that isomiR’s expression to the total expression of its corresponding canonical miRNA and all of its isomiRs. Using this definition, we found that the frequencies of high-confidence 3′ adenylated isomiRs and A-to-I edited isomiRs were significantly lower in LUAD samples than ANL samples for all three cohorts (Figure 2B). There were no consistent, significant differences in the rates of A-to-I editing, adenylation, or uridylation between groups of tumors of different stages or groups of patients with different smoking histories. However, below-median rates of A-to-I editing in TCGA LUAD samples were near-significantly associated with poorer overall survival (*p* = 0.069), suggesting that the downregulation of miRNA editing in LUAD may have significant biological consequences (Figure 2C).

### 3.3. miRNA-Modifying Enzymes Are Dysregulated in LUAD

**Figure 3:**
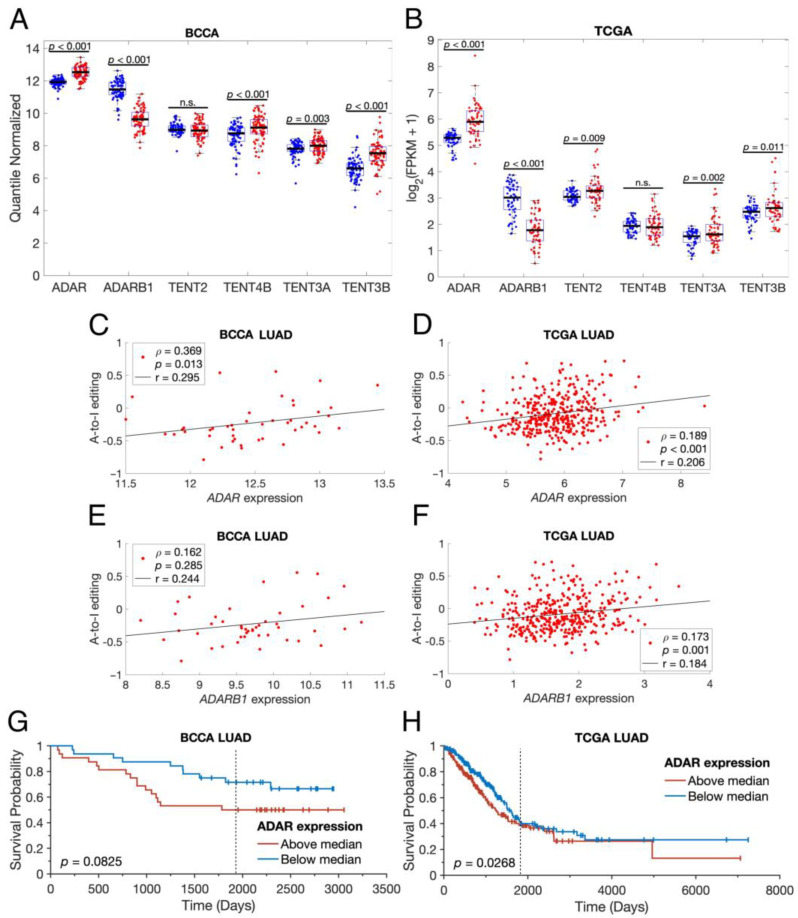
LUAD tumors alter their expression of miRNA-modifying enzymes. (**A**,**B**) Scatter-box plots showing the normalized expression of six different miRNA-modifying enzymes in ANL samples (blue points) and paired LUAD samples (red points). The displayed *p*-values resulted from paired-sample *t*-tests. n.s.: not significant, FPKM: fragments per kilobase of transcript per million mapped reads. (**C**–**F**) Scatter plots of sample-wide A-to-I editing rates vs. normalized ADAR or ADARB1 expression, where each point represents a single LUAD sample. mRNA expression normalization is identical to that shown in (**A**,**B**). Black lines are least-squares lines of best fit. The displayed *p*-values indicate the likelihood that, in an uncorrelated system, Spearman’s ρ would be as far or farther from zero than the displayed ρ. (**G**,**H**) Overall survival curves for BCCA LUAD (*n* = 64) and TCGA LUAD (*n* = 500) patients for whom mRNA expression and clinical data were available, stratified by their tumors’ ADAR expression. The displayed *p*-values resulted from log-rank tests. The graphical representations were generated using MatSurv [47]. The vertical line indicates the five-year mark. BCCA: British Columbia Cancer Agency, LUAD: Lung Adenocarcinoma; TCGA: The Cancer Genome Atlas.

To investigate the causes of these changes to the lung isomiRome in LUAD, we examined the expression of enzymes known to modify miRNAs: ADAR and ADARB1 for A-to-I editing, TENT2 and TENT4B for 3′ adenylation, and TENT3A and TENT3B for 3′ uridylation. In both the BCCA and TCGA cohorts, ADAR, TENT3A, and TENT3B were more highly expressed in LUAD samples than paired ANL samples, while ADARB1 was less highly expressed in LUAD (Figure 3A,B). Additionally, ADAR expression was significantly positively correlated with the rate of A-to-I editing in both BCCA and TCGA LUAD samples, as was ADARB1 in TCGA LUAD (Figure 3C–F). Interestingly, the above-median expression of ADAR within LUAD samples was significantly associated with poorer overall survival in the TCGA cohort and trended towards significance in the BCCA cohort (Figure 3G,H). This is likely due to ADAR’s editing of other RNAs [48], as high rates of miRNA editing were associated with improved outcomes (Figure 2C).

### 3.4. Individual miRNAs Are Modified in Distinct Fashions in LUAD

**Figure 4:**
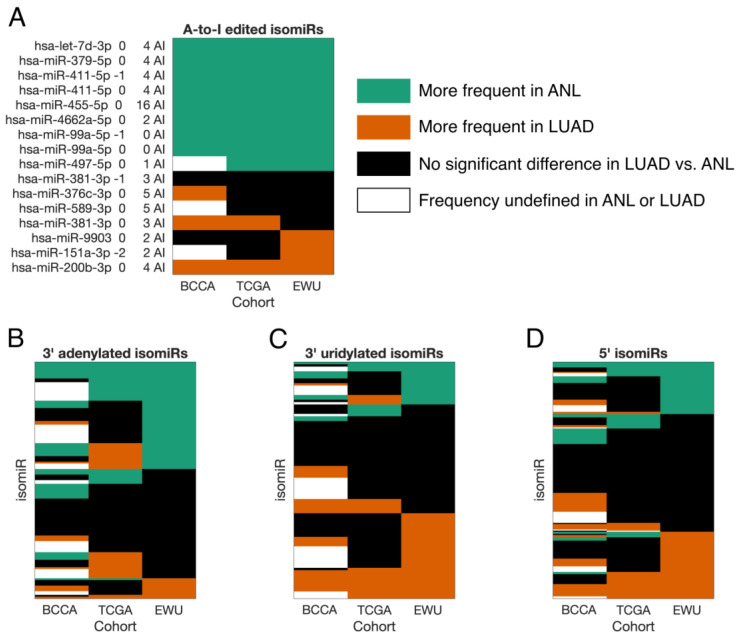
isomiR frequencies are not uniformly altered in LUAD. (**A**–**D**) Heatmaps indicating whether the frequencies of individual high-confidence isomiRs (each represented by a row) were significantly altered in LUAD vs. ANL for each of the three adult lung cohorts (represented by columns). Significance was determined by paired-sample *t*-tests with a threshold of a BH-corrected *p*-value < 0.05. White boxes, as indicated in the top-right legend, represent isomiRs whose frequencies were undefined in the ANL or LUAD samples of a particular cohort due to their corresponding miRNAs having zero expression in every sample. ANL: Adult Non-malignant Lung; BCCA: British Columbia Cancer Agency; EWU: Ewha Womans University; LUAD: Lung Adenocarcinoma; TCGA: The Cancer Genome Atlas.

Having examined high-level differences between the ANL and LUAD isomiRomes, we next investigated how the expression of individual high-confidence isomiRs varied between ANL and LUAD. Eight (50%) of the sixteen A-to-I edited isomiRs had significantly lower frequencies in LUAD in all three cohorts, which is consistent with LUAD’s lower overall editing rate (Figure 4A). Similarly, 36 (28%) of the 3′ adenylated isomiRs had significantly lower frequencies in LUAD in two or more cohorts, as opposed to only 7 (5%) with significantly higher frequencies (Figure 4B). The converse was true for 3′ uridylated isomiRs (23% with higher frequencies in LUAD; 9% lower) and 5′ isomiRs (19% higher; 11% lower) (Figure 4C,D).

**Figure 5:**
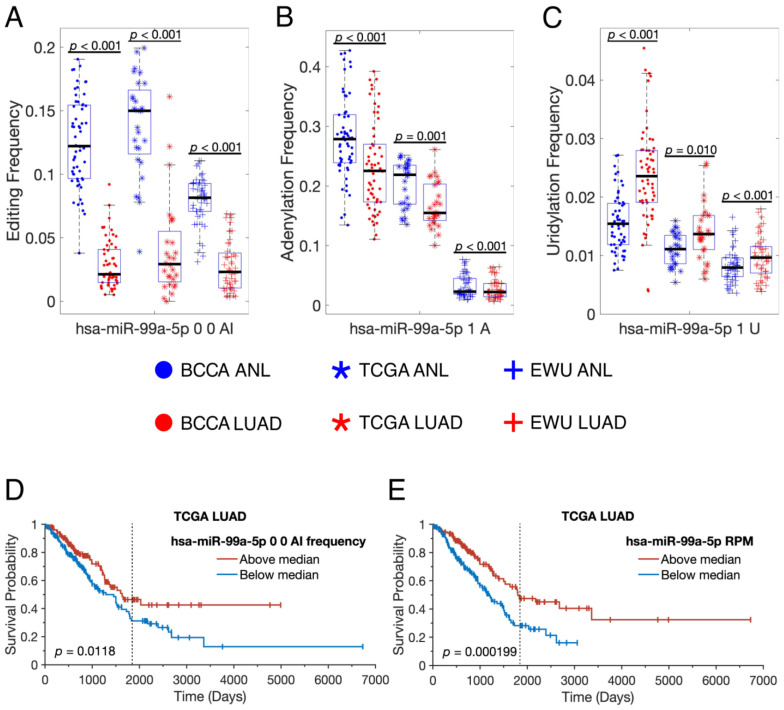
The isomiR profile of hsa-miR-99a-5p is substantially altered in LUAD. (**A**–**C**) Scatter-box plots showing the frequencies of high-confidence isomiRs of hsa-miR-99a-5p in different sample groups, which are indicated by the central legend. Each point represents a single sample, and only paired ANL and LUAD samples were included. The displayed *p*-values resulted from paired-sample *t*-tests. (**D**,**E**) Overall survival curves for TCGA LUAD patients for whom small RNA sequencing and clinical data were available (*n* = 379), stratified by the frequency with which hsa-miR-99a-5p was edited (**D**) or by the RPM expression of hsa-miR-99a-5p (**E**) in their tumors. The displayed *p*-values resulted from log-rank tests. The graphical representations were generated using MatSurv [47]. The vertical line indicates the five-year mark. ANL: Adult Non-malignant Lung; BCCA: British Columbia Cancer Agency; EWU: Ewha Womans University; LUAD: Lung Adenocarcinoma; TCGA: The Cancer Genome Atlas.

To provide an example of how individual miRNAs are modified differently in LUAD, we decided to look at the isomiRs of hsa-miR-99a-5p in more detail, as that miRNA has high-confidence sites of A-to-I editing, 3′ adenylation, and 3′ uridylation. In all three cohorts, hsa-miR-99a-5p was edited less frequently, adenylated less frequently, and uridylated more frequently in LUAD than in ANL (Figure 5A–C). As a result, the overall isomiR profile of hsa-miR-99a-5p, which could impact its mRNA targets and stability, was dramatically altered in LUAD. Higher editing of hsa-miR-99a-5p was associated with prolonged survival in the TCGA cohort, suggesting that the edited isomiR may collaborate with the unedited miRNA, which was also associated with prolonged survival and is a known tumor suppressor (Figure 5D,E) [49].

**Figure 6:**
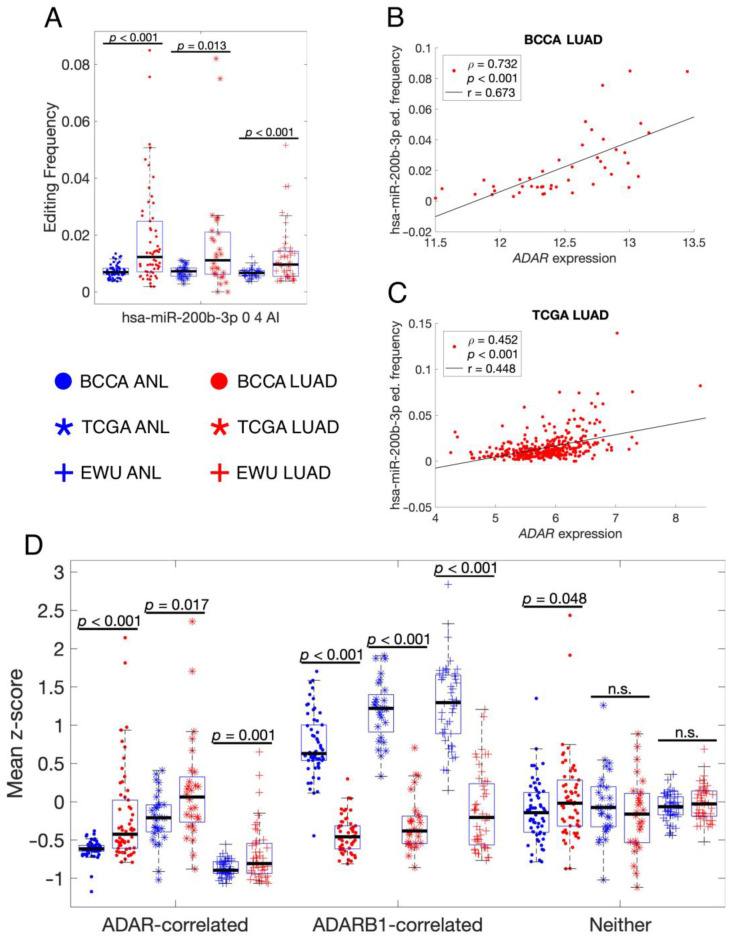
Only ADARB1-correlated edited isomiRs are less frequent in LUAD vs. ANL. (**A**) Scatter-box plots showing the editing frequency of hsa-miR-200b-3p 0 4 AI in different sample groups, which are indicated by the central legend. Each point represents a single sample, and only paired ANL and LUAD samples were included. The displayed *p*-values resulted from paired-sample *t*-tests. (**B**,**C**) Scatter plots of hsa-miR-200b-3p 0 4 AI editing frequencies vs. ADAR expression, where each point represents a single LUAD sample. Black lines are least-squares lines of best fit. The displayed *p*-values indicate the likelihood that, in an uncorrelated system, Spearman’s ρ would be as far or farther from zero than the displayed ρ. ed.: editing. (**D**) Scatter-box plots showing the rates of A-to-I editing for the three groups of high-confidence edited isomiRs delineated in Table 3. The same paired samples and sample groups were included as for (**A**), and the displayed *p*-values resulted from paired-sample *t*-tests. n.s.: not significant. ANL: Adult Non-malignant Lung; BCCA: British Columbia Cancer Agency; EWU: Ewha Womans University; LUAD: Lung Adenocarcinoma; TCGA: The Cancer Genome Atlas.

A second interesting example is the A-to-I edited isomiR of hsa-miR-200b-3p, which was the only edited isomiR that was present at a significantly higher frequency in LUAD vs. ANL for all three cohorts (Figure 6A). This suggests that hsa-miR-200b-3p was mainly edited by ADAR, which was also upregulated in LUAD (Figure 6A,B). Indeed, hsa-miR-200b-3p’s editing frequency was significantly correlated with ADAR expression in both BCCA and TCGA LUAD samples (Figure 6B,C). In contrast, the frequency of most other edited isomiRs instead correlated significantly with ADARB1 expression (Table 3). When grouping isomiRs by whether their editing frequency in TCGA LUAD was positively correlated with ADAR, ADARB1, or neither gene, only the ADARB1 group showed a decline in editing in LUAD (Figure 6D). This suggests that the overall decline in editing in LUAD, which is associated with poorer patient outcomes (Figure 2C), is attributable to the decline in ADARB1 expression.

### 3.5. IsomiR-Based Biomarkers Distinguish ANL and LUAD Samples

Based on the significant differences observed between the ANL and LUAD isomiRomes, we created SVM and RF classifiers to see if isomiRs could differentiate between ANL and LUAD samples from the same cohort. Each classifier was trained on either RPM or frequency data for one type of isomiR or on miRNA RPM data. Out of the 48 isomiR-based classifiers, 44 (92%) were found to be highly accurate (classification error < 5%) in distinguishing ANL and LUAD samples (Table 4).

**Figure 7:**
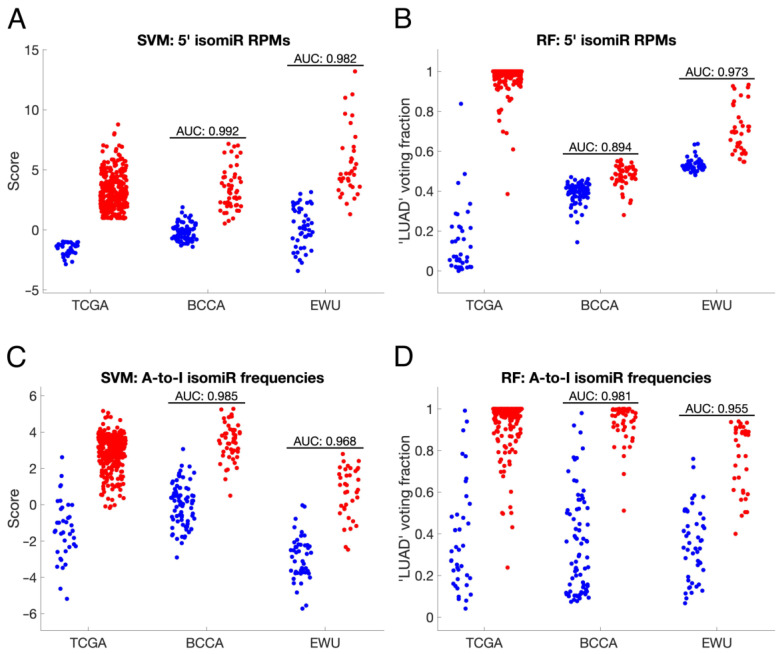
IsomiR-based classifiers accurately distinguish ANL and LUAD samples. (**A**–**D**) Plots of SVM scores and RF voting fractions for the training cohort (TCGA) and test cohorts (BCCA and EWU). Blue points represent ANL samples, and red points represent stage I/II LUAD samples. Each classifier was trained on either RPM or frequency data for one type of isomiR, as outlined in each panel’s title. SVM and RF hyperparameters were optimized prior to testing, as described in the methodology. AUC: Area Under the Receiver Operating Characteristic Curve; BCCA: British Columbia Cancer Agency; EWU: Ewha Womans University; RF: Random Forest; RPM: Reads Per Million; SVM: Support Vector Machine; TCGA: The Cancer Genome Atlas.

To test the generalizability of isomiR-based biomarkers, we trained a second batch of classifiers on either RPM or frequency data for one type of isomiR each, using only TCGA cohort samples, and optimized their hyperparameters through 5-fold cross-validation (CV). Classifiers trained on the RPM values of 5′, A-to-I edited, or 3′ uridylated isomiRs were highly accurate when tested on the BCCA and EWU cohorts (mean AUCs of 0.949–0.977) (Table 5 and Figure 7A,B). The classifier trained on the frequency of A-to-I edited isomiRs achieved a similar level of accuracy (mean AUC = 0.972) and compared favorably to classifiers trained on miRNA expression alone (mean AUC = 0.946) (Table 5 and Figure 7C,D). However, the optimal decision threshold for most of these classifiers varied considerably between cohorts (Figure 7A–D).

## 4. Discussion

In this study, we analyzed the expression of miRNAs and four major types of isomiRs in 163 ANL and 506 LUAD samples from three independent human lung cohorts. To the best of our knowledge, this is the most comprehensive analysis of isomiR expression in the human lung to date and the first study to investigate the expression of multiple types of isomiRs across multiple lung cohorts. The extensive scope of this approach led to the discovery that the expression patterns of these four types of isomiRs are altered in LUAD, making the LUAD isomiRome unique.

We detected the expression of 16 A-to-I edited isomiRs, 213 5′ isomiRs, 128 3′ adenylated isomiRs, 100 3′ uridylated isomiRs, and 654 miRNAs in multiple cohorts. In total, isomiRs made up 41% of the unique high-confidence sequences. Not all of these isomiRs will necessarily target different mRNAs than their canonical miRNAs, but this abundance suggests that regulation of the rate of miRNA modifications is a viable mechanism through which tumors can dysregulate core cellular pathways. Indeed, some of the isomiRs found to be deregulated in this study, such as hsa-miR-200b-3p 0 4 AI and the 5′ isomiR hsa-miR-140-3p 1, have been shown to have distinct targets from their canonical miRNAs and to thereby influence cancer cell viability and migration [22,26].

Upon comparing the ANL and LUAD isomiRomes, we found that many isomiRs of each of the four types were expressed at significantly different frequencies, relative to their canonical miRNAs, in LUAD vs. ANL. Decreases in miRNA editing are an established phenomenon in many forms of cancer [21], but we found that the overall rate of 3′ adenylation also decreased in LUAD. This change may significantly impact the functional lung miRNome, as 3′ adenylation has been shown to diminish miRNA-AGO2 interaction and could also alter a miRNA’s 3′-region complementarity with miRNA response elements (MREs) [29]. There was also a trend towards prolonged survival in patients with higher overall miRNA editing rates, which is consistent with the past identification of several edited isomiRs as tumor suppressors [10].

Interestingly, the respective upregulation and downregulation of the ADAR and ADARB1 editing enzymes in LUAD appeared to cause corresponding changes in editing rates: ADARB1-correlated isomiRs, which were a majority, were edited less in LUAD, and the small number of ADAR-correlated isomiRs were more highly edited in LUAD. Yet, despite miRNA editing rates tending to be lower in patients who experienced poor outcomes, low ADAR expression was linked to improved outcomes. This negative impact of ADAR is thought to be due to its editing of other families of RNAs [50]. For example, ADAR can promote LUAD cell migration and invasion by stabilizing the FAK mRNA through A-to-I editing [48]. Future studies should explore the relationship between mRNA expression changes and corresponding protein levels to fully understand their implications for cellular functions and outcomes.

The influence of adenylation and uridylation enzymes on the lung isomiRome was less clear, as rates of adenylation declined in LUAD even though the expression of adenylation enzymes was not consistently altered, and rates of uridylation were not consistently altered even though uridylation enzymes were upregulated in LUAD. One potential explanation for this incongruence is that RNA sequencing takes a snapshot of the transcriptome at a single point in time, and 3′ NTAs can significantly alter miRNA stability [28,51]. The observed rate of adenylation/uridylation for miRNAs whose stability is modulated by the NTA will thus vary with the expression and activity of exoribonucleases, and isomiRs whose NTAs mark them for imminent degradation may go undetected altogether. In addition to this scenario, it is also possible that miRNA adenylation and uridylation in the lung are substantially influenced by enzymes other than the ones investigated here, such as TENT1 and TENT6 [52].

Finally, SVM and RF classifiers trained on either isomiR RPM or frequency values demonstrated high accuracy in distinguishing ANL from stage I/II LUAD samples, even when applied to cohorts different from those they were trained on. Notably, classifiers trained on the RPM or frequency of A-to-I edited isomiRs achieved mean test cohort AUCs of 0.977 and 0.972, respectively, using only seven features each. These classifiers outperformed a previously published statistical model based on miRNA editing frequencies, which achieved a mean AUC of 0.895 upon cross-validation and was not tested in other cohorts [21]. A limitation of most of these classifiers is that the optimal score cutoff varied substantially between cohorts. This variance could be attributed to differences in sample preparation and sequencing methods (e.g., microdissection, Illumina HiSeq2000 vs. 2500) or patient characteristics (e.g., gender, race, and smoking history) across the three cohorts. Ultimately, isomiR-based biomarkers hold promise for the diagnosis of early-stage lung adenocarcinoma if they are measurable from liquid biopsy samples and show consistency across patients. Encouragingly, previous studies have identified isomiRs in serum, with an overlap observed between lung tissue-edited and serum-edited isomiRs [23,53,54].

## 5. Conclusions

This study provides a comprehensive analysis of isomiR expression across various lung cohorts, offering an extensive overview of multiple isomiR types in the human lung. Our findings suggest that ANL and LUAD tissues have highly diverse isomiRomes. The deregulation of miRNA-modifying enzymes in LUAD contributes to both global and miRNA-specific differences between these isomiRomes, presenting diagnostic opportunities and potentially influencing coding gene regulation in tumors. Future studies may shed light on the function of these isomiRs, as well as the specific mRNAs that they regulate, potentially leading to the discovery of new therapeutic targets.

## Figures and Tables

**Table 1:** Clinical characteristics of patients from the three lung adenocarcinoma (LUAD) cohorts for small RNA sequence analyses.

Characteristic	EWU (*n* = 48)	TCGA (*n* = 389)	BCCA (*n* = 63)
Median Age (Range)	59.5 (37–78)	66 (39–88)	70 (45–86)
Sex			
Male	0 (0%)	173 (44%)	19 (30%)
Female	48 (100%)	216 (56%)	44 (70%)
Smoking History			
Current or Former	7 (15%)	307 (79%)	40 (63%)
Never	41 (85%)	64 (16%)	23 (37%)
Stage			
IA	23 (48%)	102 (26%)	23 (37%)
IB	7 (15%)	103 (26%)	18 (29%)
IIA	5 (10%)	43 (11%)	2 (3%)
IIB	1 (2%)	52 (13%)	11 (17%)
IIIA	12 (25%)	57 (15%)	4 (6%)
IIIB	0 (0%)	6 (2%)	1 (2%)
IV	0 (0%)	17 (4%)	1 (2%)

Legend: BCCA—British Columbia Cancer Agency; EWU—Ewha Womans University; TCGA—The Cancer Genome Atlas; *n*—number.

**Table 2:** List of high-confidence A-to-I edited isomiRs in the human lung.

miRNA	5′ Shift	Edited Position	Adjacent Nucleotides *
hsa-let-7d-3p	0	4	U**A**C
hsa-miR-151a-3p	−2	2	U**A**G
hsa-miR-200b-3p	0	4	U**A**C
hsa-miR-376c-3p	0	5	U**A**G
hsa-miR-379-5p	0	4	U**A**G
hsa-miR-381-3p	−1	3	U**A**C
hsa-miR-381-3p	0	3	U**A**C
hsa-miR-411-5p	−1	4	U**A**G
hsa-miR-411-5p	0	4	U**A**G
hsa-miR-455-5p	0	16	U**A**C
hsa-miR-4662a-5p	0	2	U**A**G
hsa-miR-497-5p	0	1	C**A**G
hsa-miR-589-3p	0	5	A**A**C
hsa-miR-9903	0	2	U**A**U
hsa-miR-99a-5p	−1	0	A**A**A
hsa-miR-99a-5p	0	0	A**A**A

Legend: * The nucleotides immediately 5′ and 3′ of the edited adenosine (bolded) are listed. Underlined nucleotides were present only in the pri- or pre-miRNA and not in the mature canonical miRNA. Both the edited and unedited versions of the miRNAs were detected in the sequence reads.

**Table 3:** Correlations of isomiR editing rates with ADAR/ADARB1 expression.

Edited isomiR	ADAR ρ Value	ADAR *p*-Value	ADARB1 ρ Value	ADARB1 *p*-Value	Group *
hsa-let-7d-3p 0 4 AI	0.079	0.124	0.198	9.38 × 10^−5^	ADARB1
hsa-miR-151a-3p -2 2 AI	0.267	1.12 × 10^−7^	0.067	0.192	ADAR
hsa-miR-200b-3p 0 4 AI	0.452	9.65 × 10^−21^	−0.040	0.436	ADAR
hsa-miR-376c-3p 0 5 AI	−0.031	0.547	0.030	0.559	Neither
hsa-miR-379-5p 0 4 AI	−0.030	0.561	0.249	7.63 × 10^−7^	ADARB1
hsa-miR-381-3p -1 3 AI	−0.070	0.170	0.006	0.904	Neither
hsa-miR-381-3p 0 3 AI	0.061	0.233	−0.118	0.021	Neither
hsa-miR-411-5p -1 4 AI	−0.047	0.357	0.103	0.044	Neither
hsa-miR-411-5p 0 4 AI	0.008	0.874	0.171	7.97 × 10^−4^	ADARB1
hsa-miR-455-5p 0 16 AI	−0.030	0.559	0.268	9.75 × 10^−8^	ADARB1
hsa-miR-4662a-5p 0 2 AI	−0.017	0.745	0.224	9.28 × 10^−6^	ADARB1
hsa-miR-497-5p 0 1 AI	−0.046	0.374	0.233	3.80 × 10^−6^	ADARB1
hsa-miR-589-3p 0 5 AI	0.319	1.52 × 10^−10^	−0.027	0.592	ADAR
hsa-miR-9903 0 2 AI	−0.052	0.343	−0.095	0.081	Neither
hsa-miR-99a-5p -1 0 AI	−0.135	0.008	0.116	0.023	ADARB1
hsa-miR-99a-5p 0 0 AI	−0.081	0.114	0.310	5.48 × 10^−10^	ADARB1

Legend: Only TCGA LUAD samples were included in the correlation analysis. The displayed *p*-values indicate the likelihood that, in an uncorrelated system, Spearman’s ρ would be as far or farther from zero than the displayed ρ. * isomiRs were assigned to the “Neither” group unless they had a positive correlation with ADAR or ADARB1 expression that was significant after BH correction (FDR < 0.05).

**Table 4:** Error rates for single-cohort miRNA and isomiR-based classifiers.

		RPM-Based Classifiers					Frequency-Based Classifiers			
		miRNA	3′ U	3′ A	A-to-I	5′	3′ U	3′ A	A-to-I	5′
**Mean classification error in 10-fold CV**	BCCA SVM	1.56%	1.56%	0.78%	3.13%	2.34%	0.78%	0.00%	4.69%	3.13%
	TCGA SVM	1.45%	1.16%	2.03%	1.45%	0.58%	1.16%	2.62%	1.74%	1.16%
	EWU SVM	2.38%	2.38%	2.38%	2.38%	2.38%	1.19%	3.57%	11.90%	3.57%
	Mean	1.80%	1.70%	1.73%	2.32%	1.77%	1.04%	2.06%	6.11%	2.62%
**Out of bag error**	BCCA RF	0.78%	0.78%	0.00%	1.56%	0.78%	4.69%	1.56%	3.91%	4.69%
	TCGA RF	1.45%	0.58%	0.87%	1.16%	0.87%	1.45%	2.62%	2.62%	1.74%
	EWU RF	1.19%	0.00%	0.00%	1.19%	0.00%	8.33%	14.29%	3.57%	5.95%
	Mean	1.14%	0.45%	0.29%	1.30%	0.55%	4.82%	6.16%	3.37%	4.13%

Legend: ANL—Adult Non-malignant Lung; BCCA—British Columbia Cancer Agency; EWU—Ewha Womans University; LUAD—Lung Adenocarcinoma; RF—Random Forest; RPM—Reads Per Million; SVM—Support Vector Machine; TCGA—The Cancer Genome Atlas.

**Table 5:** Test cohort AUCs for TCGA-trained miRNA and isomiR-based classifiers.

		RPM-Based Classifiers					Frequency-Based Classifiers			
		miRNA	3′ U	3′ A	A-to-I	5′	3′ U	3′ A	A-to-I	5′
Test Cohort AUCs	Features	368	37	41	7	115	17	34	7	36
	BCCA SVM	0.944	0.998	0.962	0.997	0.992	0.663	0.476	0.985	0.716
	EWU SVM	0.951	0.995	0.884	0.934	0.982	0.582	0.752	0.968	0.893
	BCCA RF	0.901	0.891	0.855	0.990	0.894	0.634	0.756	0.981	0.889
	EWU RF	0.988	0.910	0.995	0.988	0.973	0.572	0.704	0.955	0.606
	Mean	0.946	0.949	0.924	0.977	0.960	0.613	0.672	0.972	0.776

Legend: AUC—Area Under the Receiver Operating Characteristic Curve; BCCA—British Columbia Cancer Agency; EWU—Ewha Womans University; LUAD—Lung Adenocarcinoma; RF—Random Forest; RPM—Reads Per Million; SVM—Support Vector Machine; TCGA—The Cancer Genome Atlas.

## Data Availability

The datasets supporting the findings of this study are publicly available as follows: miRNA sequencing data for the TCGA cohort were retrieved from Genomic Data Commons Data Portal (https://portal.gdc.cancer.gov/, accessed on 11 July 2019). mRNA expression data and patient clinical information, including survival data, were obtained from the University of California Santa Cruz (UCSC) Xena browser (https://xenabrowser.net/, accessed on 11 July 2019). miRNA sequencing data for the Ewha Womans University (EWU) cohort were downloaded from the European Nucleotide Archive (Study Accession: PRJNA434883) (https://www.ebi.ac.uk/ena/browser/home, accessed on 12 February 2021). Clinical data for the EWU cohort were acquired through the Sequence Read Archive run selector (SRA Accession: SRP133217) (https://www.ncbi.nlm.nih.gov/sra, accessed on 12 February 2021). miRNA expression data for samples in the BCCA cohort were deposited in the National Center for Biotechnology Information Gene Expression Omnibus (GEO accession: GSE175462).

## Supplementary Materials

The following supporting information can be downloaded at: https://www.mdpi.com/article/10.3390/cancers16193322/s1, Figure S1: Biogenesis and functional consequences of selected miRNA modifications.
