# Supplementary material for: Post-Transcriptional Modifications to miRNAs Undergo Widespread Alterations, Creating a Unique Lung Adenocarcinoma IsomiRome
Source: Cancers (Basel). 2024 Sep 28;16(19):3322. doi: 10.3390/cancers16193322 (PMC11476290; doi:10.3390/cancers16193322)
Supplement: Supplementary file 1 [file cancers-16-03322-s001.zip › cancers-3210163-supplementary.pdf]

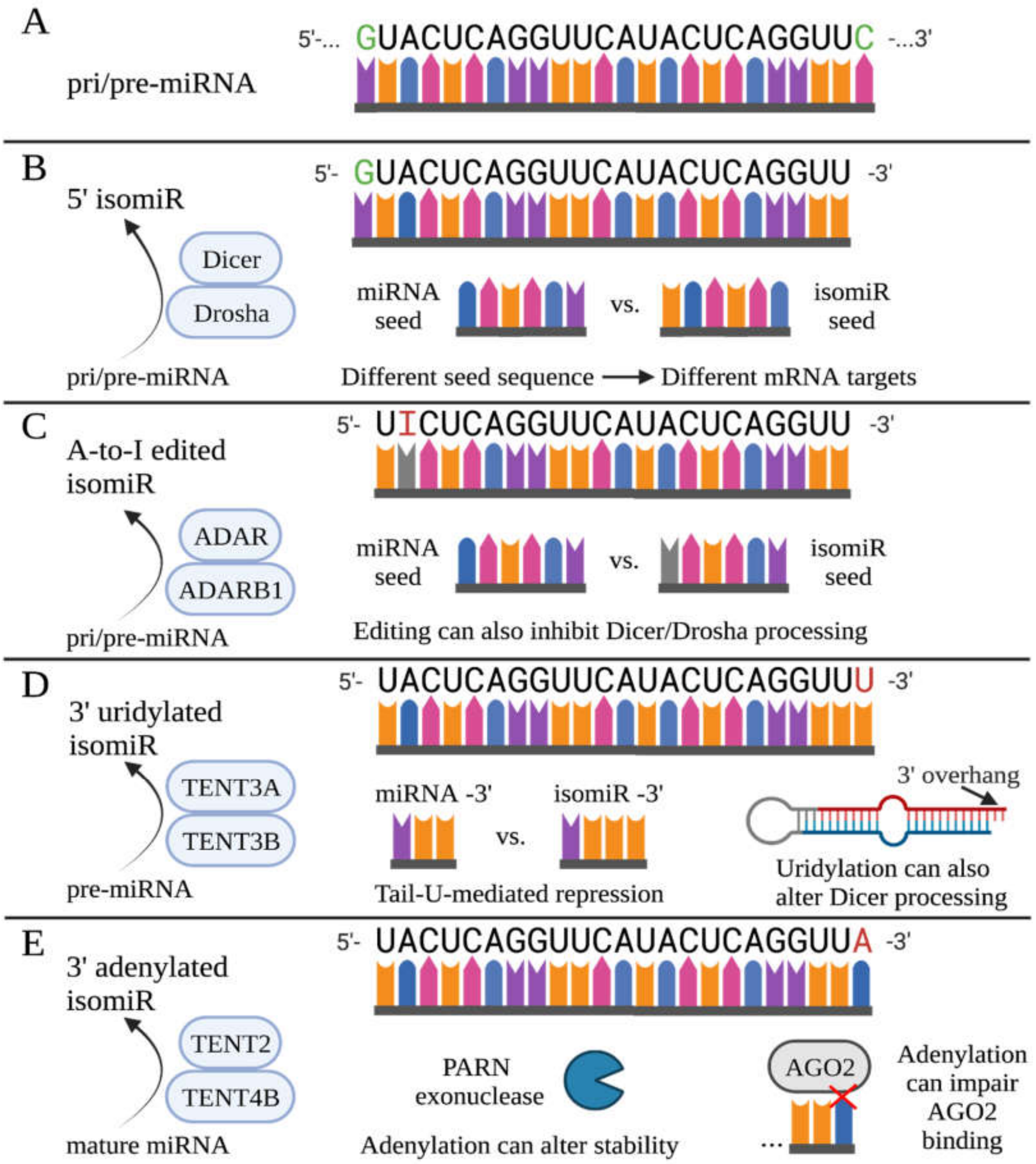

Figure S1. Biogenesis and functional consequences of selected miRNA modifications. (**A**) An example pri/pre-miRNA sequence, provided for comparison with the mature isomiR sequences in (**B-E**); (**B**) The shifted seed sequence of a 5′ isomiR typically grants it mRNA targets that are distinct from those of its canonical miRNA; (**C**) A-to-I editing can impair Dicer or Drosha processing, and, when occurring in seed regions, significantly alters mRNA targeting specificity; (**D**) 3′ uridylation can modulate Dicer processing by altering the length of a pre-miRNA's 3′ overhang. In mature isomiRs, the extra uracil nucleotide can expand the isomiR's mRNA target repertoire through tail-U-mediated repression; (**E**) 3′ adenylation can impact an isomiR's gene silencing activity by modifying its susceptibility to exonucleases or limiting its capacity to bind AGO2. For all panels, the schematics on the left-hand side indicate the primary miRNA-modifying enzymes, along with their substrate(s). In the nucleotide sequences at the top-middle of each panel, black letters correspond to nucleotides present in the canonical mature miRNA, green letters correspond to nucleotides normally present only in the pri- or pre-miRNA, and red letters correspond to non-templated nucleotides (i.e. those not present in the miRNA gene). Created with BioRender.com.
